# Design and Validation of a Monte Carlo Method for the Implementation of Noninvasive Wearable Devices for HbA1c Estimation Considering the Skin Effect

**DOI:** 10.3390/mi15091067

**Published:** 2024-08-24

**Authors:** Tae-Ho Kwon, Shifat Hossain, Mrinmoy Sarker Turja, Ki-Doo Kim

**Affiliations:** 1 Department of Electronics Engineering, Kookmin University, Seoul 02707, Republic of Korea; kmjkth@kookmin.ac.kr (T.-H.K.); mrinmoyturja@kookmin.ac.kr (M.S.T.); 2 Department of Electrical and Computer Engineering, University of Central Florida, Orlando, FL 32816, USA; shifathosn@gmail.com

**Keywords:** glycated hemoglobin (HbA1c), noninvasive, PPG signals, skin effect, Monte Carlo method, wearable device

## Abstract

To diagnose diabetes early or to maintain stable blood glucose levels in diabetics, blood glucose levels should be frequently checked. However, the only way to check blood glucose levels regularly is to use invasive methods, such as pricking the fingertip or using a minimally invasive patch. These invasive methods pose several problems, including being painful and potentially causing secondary infections. This study focuses on noninvasively measuring glycated hemoglobin (HbA1c) using PPG signals. In particular, the study relates to a method and a hardware design technology for removing noise that may be present in a PPG signal due to skin contact with a noninvasive HbA1c measurement device. The proposed HbA1c measurement device consists of the first sensor (PPG sensor) module including an optical barrier and the second sensor (cylindrical sensor) module for removing the skin effect. We have developed a Monte Carlo method to implement accurate, noninvasive HbA1c measurement by considering different skin properties among different subjects. Implementing this model in wearable devices will allow end users to not only monitor their glycated hemoglobin levels but also control diabetes with higher accuracy without needing any blood samples. This will be a groundbreaking advancement in modern wearable medical devices.

## 1. Introduction

Diabetes is a disease in which there is an abnormality in the secretion or function of insulin, which is necessary for controlling blood glucose levels in the body. The glycated hemoglobin (HbA1c) test is used to determine how much hemoglobin in red blood cells is glycated and reflects changes in blood glucose over the past 3 months according to the average lifespan of red blood cells. People with diabetes can reduce the rate of worsening or complications of diabetes through glycemic control, weight loss, and medication. Therefore, diabetic patients must frequently measure their blood glucose levels to manage them and must regularly undergo HbA1c testing, which is a treatment index as important as blood glucose level for diabetic patients [1].

In a conventional method for measuring HbA1c, blood is collected from a vein in the arm of a subject or a capillary blood sample is obtained by piercing the tip of a finger with a small, sharp needle. This invasive method of measuring glycated hemoglobin increases the burden of blood collection on the measurement subject and has the problem of providing inaccurate values in cases such as short red blood cell lifespan, pregnancy, kidney disease, etc. This has led to a growing interest in noninvasive and continuous monitoring techniques that can be easily integrated into patients’ daily lives [2]. Recent advances in noninvasive sensors are poised to revolutionize diabetes diagnosis and management. Various approaches, including optical methods such as near-infrared (NIR) spectroscopy and Raman spectroscopy, electromagnetic sensing, transdermal sensing, and the use of alternative biofluids such as saliva, sweat, or tears for glucose measurement, have shown some promise [3,4,5,6]. Optical methods rely on light interaction with glucose molecules but face challenges from strong background noise, requiring sophisticated algorithms for accuracy. Electromagnetic sensing measures tissue dielectric properties but is hindered by tissue heterogeneity and individual variability. One study has investigated how to measure HbA1c using acetone levels in breath [7]. Although this method is a noninvasive method, there is a risk of infection because the device must be kept close to the mouth to measure breath. Another study proposed a method for measuring HbA1c in vitro using optical sensors, but this method has the disadvantage of requiring an invasive step as blood must first be transferred to a test tube [8]. After that, one promising approach was proposed using wrist photoplethysmography (PPG) data, which measures changes in blood volume in the microvascular bed of tissue via light-emitting diodes (LEDs) and a photodetector (PD).

In our previous study [9], we devised a noninvasive method to estimate the percentage of the in vivo HbA1c values from Monte Carlo photon propagation simulations based on wrist or finger models using 3D magnetic resonance (MR) image data. However, skin effects were not considered in [9]. In this study, to estimate HbA1c noninvasively, we developed a Monte Carlo based model considering different skin properties and worked to optimize and implement the model into a low-resource wearable device. The contributions of this study, including the advantages of the proposed method, are as follows.

- Intrinsic and extrinsic noise can be removed by the model (hardware design) itself. Hence, the model will have more accurate estimates of our target parameters.
- Skin pigments (melanin and bilirubin) and the skin reflection coefficient are also considered in the epidermis of the wrist model.
- A simple sensor (cylindrical type LED-PD pair) was added to compensate for the skin effect.
- Calibration can be made simpler due to the Monte Carlo-based model considering different skin properties.
- The performance of the proposed method was demonstrated by increasing the number of subjects to 50.

## 2. Methodology

### 2.1. Skin Effect Analysis of a PPG Sensor (PPG Signal Impurities)

Upon rigorous investigation of the recorded PPG signals, we found several points that added impurities to the recorded signals.

**Figure 1:**
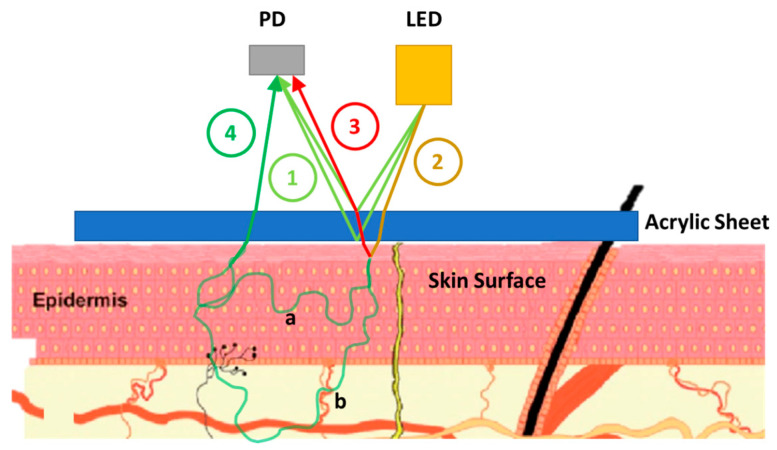
PPG signal impurities in a PPG sensor.

Figure 1 shows the diagram of the noise (impurity) sources in a PPG sensor.

Since there is no partition between the LED and PD, and there is an acrylic sheet, the intrinsic reflection of ① in Figure 1 contributes to the received signal. We denote this intrinsic component as $I_{in}$. ② in Figure 1 shows the light rays passing through an acrylic sheet and reaching the skin (e.g., the wrist). In the figure, the escaped light rays ② interact with the skin surface and two events occur. One is the direct reflection from the surface, ③ in the figure. This can be defined as the extrinsic reflection component of the received signal. We denote this component as $I_{ex}$. The other part ④ in the figure is the expected part of the light rays, which travels through the skin and interacts with the blood and non-blood components according to the theoretical model. Both scattering paths ‘a’ and ‘b’ are the paths where melanin can interact with the light intensity. However, only path ‘b’ interacts with blood components in the skin.

As mentioned above, the signals from ① and ③ show the signals from intrinsic and extrinsic reflections, respectively. The component ③ is not easy to separate as the intrinsic reflection ①. Because this reflection depends on the skin reflectivity, $R_{skin}$, the signal ③ is a function of wavelength λ and ethnicity (more specifically melanin (Mel) and bilirubin (Bil)). Therefore, we can write the extrinsic reflection component as $I_{ex}\;(\lambda ,\;Mel,\;Bil)$. The signal from ④ corresponds to the skin effect signal, which contains the expected noise-free signal ($I_{4}$) but in a modulated form ($I_{Mel}\left(\alpha \left(\lambda \right),I_{4}\right)$). We then indicate an overall equation of the received light considering intrinsic reflection, extrinsic reflection, and skin effect signals.

(1) $$I_{received}=I_{in}+I_{ex}\left(\lambda ,Mel,Bil\right)+I_{Mel}\left(\alpha \left(\lambda \right),I_{4}\right)$$

Now, if we think about it analytically, the intrinsic term ($I_{in}$) can be easily purified. Though the intrinsic reflection intensity is device specific, it is consistent over time. Therefore, this reflection can be easily removed by subtracting the reflection values from the received intensity. However, the extrinsic reflection ($I_{ex}$) and skin effect ($I_{Mel}$) terms have complicated definitions and it is very difficult to build models for each of these components.

From an analytical perspective, Equation (1) is very difficult to solve for the expected noise-free signal component ($I_{4}$). From the equation itself, we can see that the noise compensation of the received signal ($I_{received}$) is not a straightforward subtraction of the signal, since the expected noise-free signal is inside the function of the skin effect ($I_{Mel}$). For this reason, we do not go to the analytical route of constructing models for the various components of the received signal; instead, we are searching for an alternative signal that can correspond to both the extrinsic reflection and the skin effect.

We can establish that the signal from ③ (or $I_{ex}$) is a function of skin surface color and reflectivity ($R_{skin}$). The skin surface color is again a function of melanin, bilirubin, etc. Because there are a number of studies that show the relationship between skin pigmentation and skin reflectance [10], we can establish that

(2) $$I_{ex}\approx f_{1}\left(skin\;surface\;color,\;R_{skin}\right)\approx f_{2}(Mel,\;Bil)$$

The skin effect on the received signal from ④ (or $I_{Mel})$ is again a function of skin pigmentation (i.e., melanin, bilirubin, etc.) and the noise-free signal. Hence, we can write

(3) $$I_{Mel}\approx f_{3}(Mel,\;Bil,\;I_{4})$$

Here, the blood content is not considered in the skin effect since the blood effect is already considered in $I_{4}$ [9]. We can now see that $I_{ex}$ and $I_{Mel}$ have similar sources of error in their models. However, it is impossible to simplify these two models because the nature of the models is completely different.

### 2.2. Proposed Method and Hardware Design for Purifying Impurities

**Figure 2:**
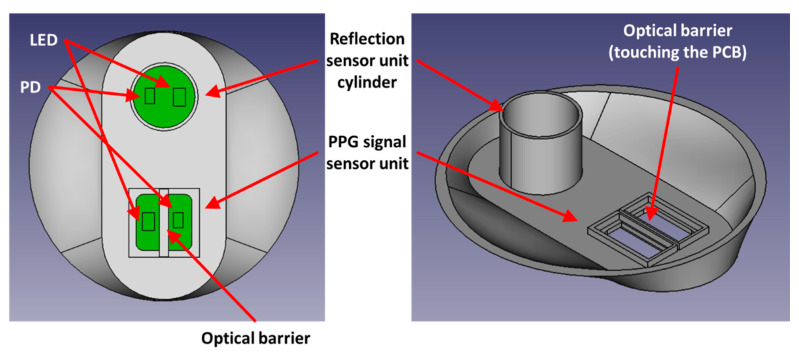
Hardware design configuration of a watch-type PPG sensor for HbA1c estimation.

First, we consider how to remove both intrinsic noise (signal ①) and extrinsic noise (signal ③). From the discussion of signal impurities, it is not easy to apply the analysis methods described above to remove extrinsic noise. Therefore, we solve this problem fundamentally by using a hardware design method. In other words, between the LED and the PD, we install an optical barrier that also separates the acrylic sheet and the PCB to eliminate most of the impurities from the source of the signal, as shown in Figure 2. Note that the optical barrier touching the skin surface blocks all of the impurities (① and ③) illustrated in Section 2.1.

Next, we propose a method to remove the skin effect from the signal ④ (or $I_{Mel})$. At this stage, we only have to find the source of the signal that can indicate the amount of melanin and bilirubin in the skin layer. The signal can then be used to inversely model the melanin and bilirubin levels again. Here, to make the skin reflection value satisfy our condition, we adopt an additional cylindrical sensor (reflection sensor) in addition to the PPG sensor, and the detailed configuration can be seen in Figure 2. Note that the optical barrier touches the PCB from the inside and it also touches the skin surface from the outside. The optical barrier is only given in the PPG sensor unit. The reflection sensor unit does not contain any barrier. The PPG sensor unit may have an acrylic sheet on top of it. However, the acrylic sheet should not cover the barrier, and the sheet must be separated by the optical barrier. The optical barrier must be in contact with the skin. An acrylic sheet is not used in the reflection sensor unit.

In the case of a cylindrical sensor, R, G, B values are captured at a certain distance from the skin surface for every person. Here, ‘certain distance’ means a distance sufficient to receive only the reflected signal from the skin surface and 17 mm was experimentally adopted in this study. Then, the R, G, B values can be said to be a function of skin pigmentation (i.e., melanin, bilirubin, etc.). This is similar to the extrinsic reflection component since the received signal at a certain distance (experimentally, 17 mm in this case) is a non-oscillating signal. Hence, it can be safely considered that the component ④ (i.e., light rays penetrating the skin) does not reach the PD after being heavily absorbed in the turbid medium. Therefore, the R, G, B skin reflection values obtained using the cylindrical sensor can be expressed as,

(4) $$\left(R,G,B\right)\approx f_{4}(Mel,Bil)$$

(5) $$g_{1}\left(R,G,B\right)\approx f_{2}\left(Mel,Bil\right)\approx I_{ex}$$

Again, there must be a solution to the melanin and bilirubin terms from the received R, G, B values.

(6) $$Mel\approx g_{2}(R,G,B)$$

(7) $$Bil\approx g_{3}(R,G,B)$$

Using $g_{2}$ and $g_{3}$, it is theoretically possible to solve $I_{Mel}$ for our expected noise-free $I_{4}$ term. Thus, even if the skin reflection R, G, B signals using the cylindrical sensor do not contain the PPG signal and therefore do not have skin effects, the skin reflection signal can theoretically be used to compensate for all of the noise sources described in the original model of the received signal.

In summary, the typical configuration of a wearable watch includes three noise sources as described in the received signal model. And the skin reflection R, G, B signals using the cylindrical sensor contain only extrinsic noise and pigment signals. Above all, it has been theoretically proven in the above description that the R, G, B signals received using a cylindrical sensor are sufficient to compensate for errors (or solve for the noise-free signal) from the received signal model. After purification of the signal, HbA1c and SpO2 estimations become much more robust and accurate.

### 2.3. Data Acquisition System

A data acquisition system was developed to record PPG signals from the wrist. Since this process of estimating HbA1c relies on at least three wavelengths of light, a white light-based PPG acquisition system was designed for simple device development.

#### 2.3.1. PPG Data Acquisition System Description

**Figure 3:**
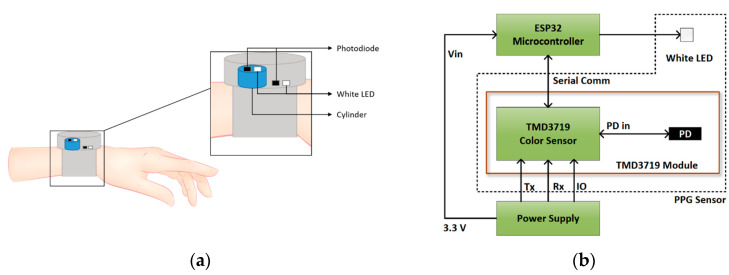
(**a**) LED and PD arrangement in the device and (**b**) block diagram of device components [11].

Figure 3a shows the process of the white LED PPG acquisition and Figure 3b shows the basic block diagram of the device [11].

The sensor module uses a TMD3719 color sensor to record light intensities at 465 nm, 525 nm, and 615 nm. The color sensor has three different filters on top of the sensor die: blue (465 nm), green (525 nm), and red (615 nm). An ESP32 microcontroller was used to communicate with the TMD 3719 module. Additionally, instead of using three high-intensity light sources with different wavelengths, only one white LED (CLM3C-WKW) was used as the light source.

Since the intensity of the PPG signal varies depending on skin color, the signal was collected with the variable gain setting, and the final signal was corrected by storing the gain information accordingly. We used a band-pass filter, and the passband was 0.5 to 8 Hz. Also, the sampling frequency used was 24 Hz because it was determined considering the maximum sampling rate of the AC to DC converter (ADC).

**Figure 4:**
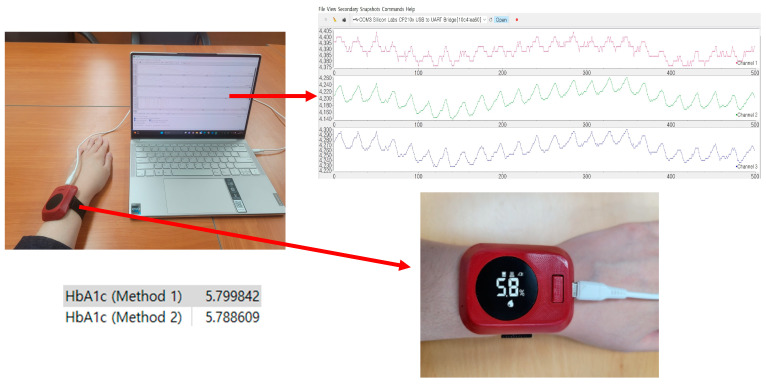
PPG data acquisition and corresponding measurement results.

Figure 4 shows an example of PPG data acquisition and a graph of the measurement results.

#### 2.3.2. PPG Data Source and Analysis

To assess the accuracy of the model and validate the methodology, an analysis was performed using a dataset consisting of a total of 28 participants under the direct supervision of the institutional review board (IRB) of Kookmin University (IRB protocol number: KMU-202111-BR-286), Seoul, Republic of Korea. The age of the participants ranged from 25 to 65 (40.0 ± 13.3) years. Of them, eleven people (39%) were pre-diabetic, six participants (22%) were diabetic, and eleven participants (39%) were healthy. Table 1 presents the statistics of the %NGSP HbA1c values. The distribution of %NGSP HbA1c values for 28 subjects and 50 subjects can be found in Supplementary Figure S1 and Figure S2, respectively. The number of subjects was set to 28 to compare the results with the results of our previously published paper [9]. As stated in Section 3.4, the number of subjects was increased to 50 to increase the reliability of the accuracy of the results, and the experimental results were compared and analyzed.

In the context of methodology, we utilized the same reference equipment as in our previous study [9]. To record the reference HbA1c and SpO2 values, we used BioHermes A1c EZ and Schiller Argus OXM Plus devices, respectively. The study process also included the recording of 2 min PPG signals. Before recording, the subjects were told to remain silent and relaxed. Since methods to reduce the influence of motion artifacts are currently being studied and we expect to have the opportunity to present the results in the near future, measurements were made in this study with the wrist as still as possible. While recording, it was also ensured that all of the subjects were provided with a comfortable environment so that they could remain relaxed.

#### 2.3.3. PPG Data Analysis

**Figure 5:**
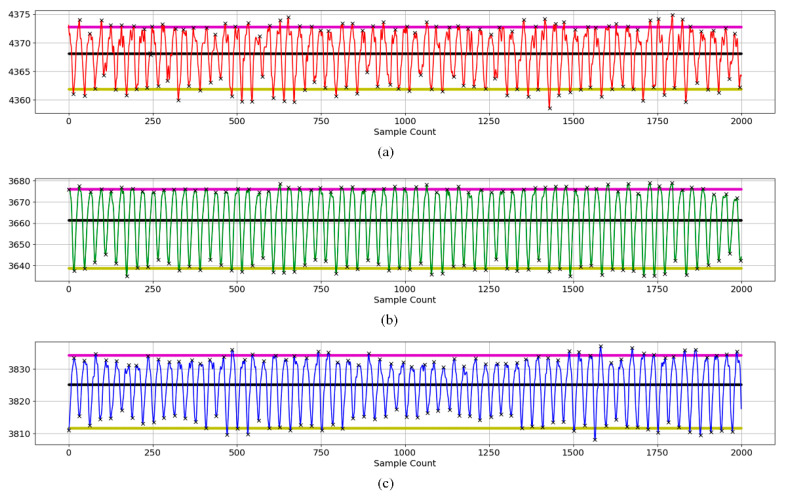
Peaks and valleys of a subject’s three-wavelength signal to determine AC and DC components (The black line represents the average value of the signal, the purple line represents the average value of the peaks, and the yellow line represents the average value of the valleys.): (**a**) red wavelength, (**b**) green wavelength, and (**c**) blue wavelength.

AC and DC values were calculated for a total of 28 subjects for three wavelengths: red, green, and blue. The AC/DC values were used as calibration inputs. An example of three wavelength PPG signals is shown in Figure 5. The black line represents the average value of the signal, the purple line represents the average value of the peaks, and the yellow line represents the average value of the valleys. The AC value is taken as the distance between the mean of the upper peaks and the mean of the lower valleys. The DC value is taken as the average of intermediate or valley values. Black x marks are used to indicate peaks and valleys.

**Figure 6:**
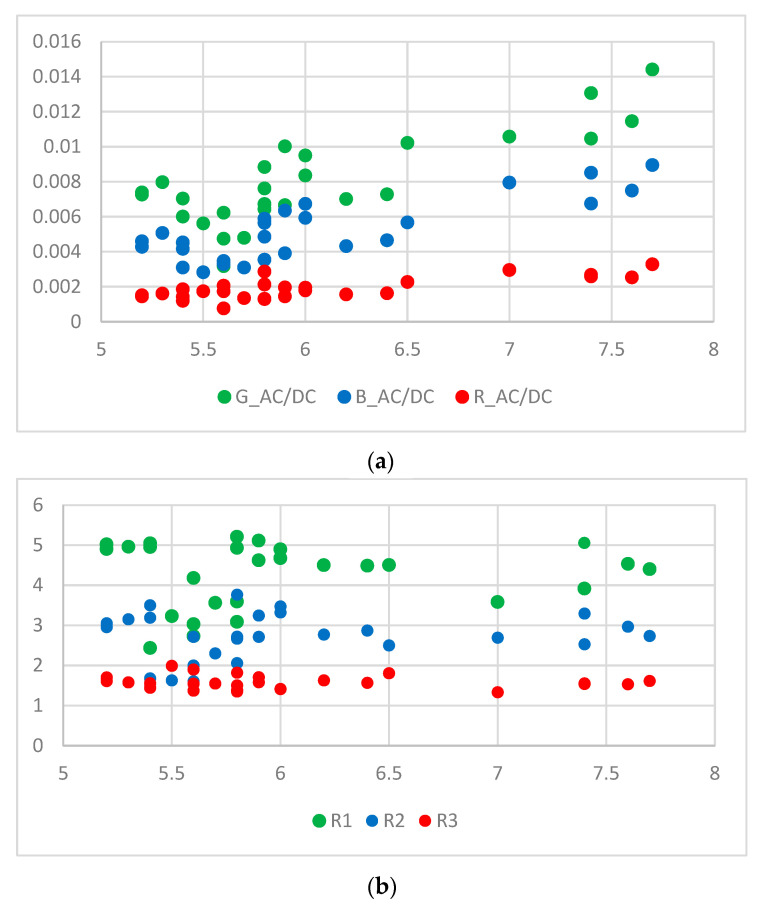
(**a**) AC/DC versus HbA1c for 28 subjects and (**b**) ratio versus HbA1c for 28 subjects.

The AC to DC ratio (AC/DC) represents the ratio of the pulsatile (i.e., AC) to the baseline or static (i.e., DC) component of the PPG signal. There could be an underlying relation between the AC/DC value and HbA1c. However, there are few studies on this issue. One study demonstrated a proportional relationship between AC/DC value and glucose [12]. Another study showed a correlation between blood glucose levels and the AC/DC values of PPG signals [13]. Figure 6 shows AC/DC values versus HbA1c and ratio versus HbA1c for 28 subjects. In the case of the green wavelength, the AC/DC value is generally larger than those of the other two wavelengths (blue and red), and the results for all three wavelengths show a tendency to roughly increase according to the HbA1c value. This tendency of AC/DC is one of the reasons for using AC/DC values as input for calibration. We may consider ratios (R1, R2, and R3) of different AC/DC values of the PPG signal as input for calibration. R1 was considered as the ratio of the green AC/DC to red AC/DC, R2 was considered as a ratio of the blue AC/DC to the red AC/DC, and R3 was considered as a ratio of green AC/DC to the blue AC/DC. These are expressed in Equations (8)–(10). The ratio does not show the same clear tendency as AC/DC. The reason is that each AC/DC used at a different ratio shows a similar tendency. However, since each R-value contains information on two AC/DCs, it can be used as valid information.

(8) $$R1=\frac{{\left[\frac{AC}{DC}\right]}_{green}}{{\left[\frac{AC}{DC}\right]}_{red}}$$

(9) $$R2=\frac{{\left[\frac{AC}{DC}\right]}_{blue}}{{\left[\frac{AC}{DC}\right]}_{red}}$$

(10) $$R3=\frac{{\left[\frac{AC}{DC}\right]}_{green}}{{\left[\frac{AC}{DC}\right]}_{blue}}$$

### 2.4. Estimation Process with Monte Carlo Simulation

#### 2.4.1. Overall Estimation Process

**Figure 7:**
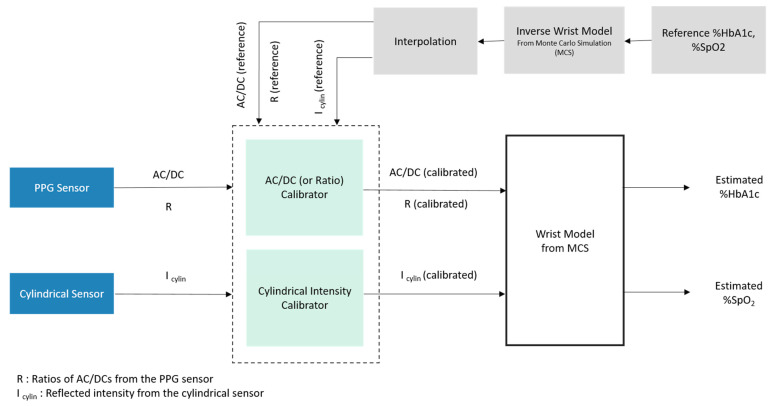
Overall estimation process diagram with the Monte Carlo simulation model.

Figure 7 shows the overall estimation process diagram. First, AC/DC values can be calculated from the input PPG signals.

The proposed sensor detects HbA1c noninvasively using PPG signals considering red, green, and blue wavelengths. The blood volume changes in the microvascular bed of tissue are detected by the PPG sensor. Therefore, after collecting the PPG signal, the AC to DC ratio (AC/DC), ratio of ratios, and cylindrical intensity values are calibrated with those of MCS. After calibration, the HbA1c level can be estimated by interpolating these calibrated values. As input to the calibrator in the PPG sensor, AC/DC values at three wavelengths and two ratios of different AC/DC values can be considered. Among these values, we applied AC/DC values at three wavelengths (R, G, B) or AC/DC values at two wavelengths (G, B) and one ratio value (R1) as calibrator input. In the latter case as a calibration input, only blue and green PPG signals were used since the red signal can be neglected because of poor quality among the wrist PPG signals. In Figure 7, the wrist model indicates obtaining HbA1c and SpO2 values from either AC/DCs or ratio (R) values. The inverse wrist model, in a similar manner, indicates obtaining either AC/DCs or ratio (R) values from the reference HbA1c and SpO2 values. Note that the wrist model and inverse wrist model are both based on Monte Carlo simulation (MCS) tables.

Two calibration models were used for calibrating AC/DCs (or ratio values) and three cylindrical signals (blue, green, and red) with the corresponding values obtained from the inverse wrist model. The target values for the calibration model were found through interpolation using the reference HbA1c and SpO2 values for each subject. For calibration, the XGBoost regression model was used for both the AC/DC (or ratio) calibrator and the cylindrical value calibrator. After calibration, the calibrated output values were aligned to the MCS table. These calibrated values were then easily interpolated with the wrist model to find the results. Estimated HbA1c and SpO2 values were then found for each subject. Every step of calibration and final estimation was observed and validated in a systematic way.

#### 2.4.2. Monte Carlo Photon Propagation Simulation

**Figure 8:**
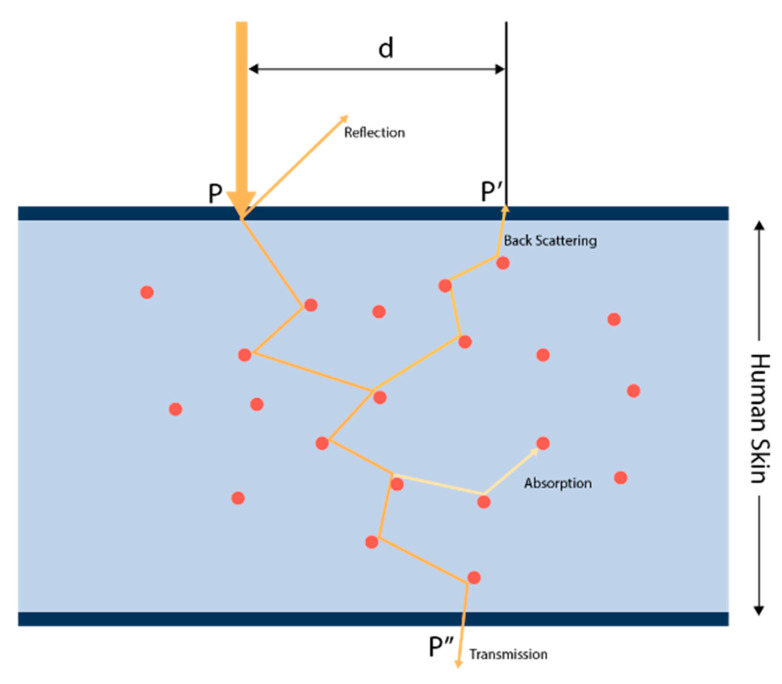
The MCS model where photons will traverse through (The red circles represent photons).

The HbA1c detection mechanism begins with using PPG signals to exploit the difference in light absorption rates with changes in HbA1c in the blood of the microvascular layer of tissue. In this study, we used Monte Carlo simulation to estimate HbA1c noninvasively. First, each layer (skin, muscle, fat, bone, blood, etc.) is identified through 3D MRI images. Here, the light absorption and scattering coefficients for each layer are calculated and applied according to changes in HbA1c, SpO2, and melanin values. We simulated the path, absorption, and scattering of the virtual photons projected onto a 3D biometric model that closely resembles reality. In this method, a number of photons are simulated through a biological/turbid media, and the received photon weights and traversing paths are recorded. To run a simulation, three elements are required: at least one source, at least one detector, and one model. The source has a position, direction, and type in the 3D space. Common types are isotropic and pencil. The detector only has a position in the 3D space. The model can be constructed by using parametric methods, voxels, meshes, etc. The mesh model contains the following properties: material–absorption coefficient (μa), scattering coefficient (μs), anisotropy factor (g), and refractive index (η). The photons will traverse through the model and then be reflected on the incident surface, where they are transmitted, absorbed, scattered, and diffusely reflected, as shown in Figure 8. In the figure, at the upper boundary of the depicted skin layer, a beam of light originates from a source external to the system. This is denoted by point P, where the light first makes contact with the skin surface. Upon this initial interaction, part of the light is reflected off the surface. As it penetrates the skin, some photons are scattered back toward the surface. This backscattering is represented by the paths that loop back up toward the point marked P′. The distance marked ‘d’ represents the distance between the incident light, P, and the photodetector, P′. As the light travels deeper into the skin, it encounters various tissue elements, symbolized by the red dots. These random tissue elements represent simplified models of the complex structures within the skin, such as cells or blood vessels. These elements absorb some of the light energy, reducing the number of photons that continue to propagate through the skin. After multiple interactions with tissue elements, including absorption and scattering, the remaining light photons reach the lower boundary of the skin. The light transmission is marked by point P″, indicating the exit of light from the skin layer. The light that is backscattered to the surface and reaches point P′ can be detected by a photodetector placed at this position. This detection can provide valuable information about the properties of the skin and the effects of light interaction.

**Figure 9:**
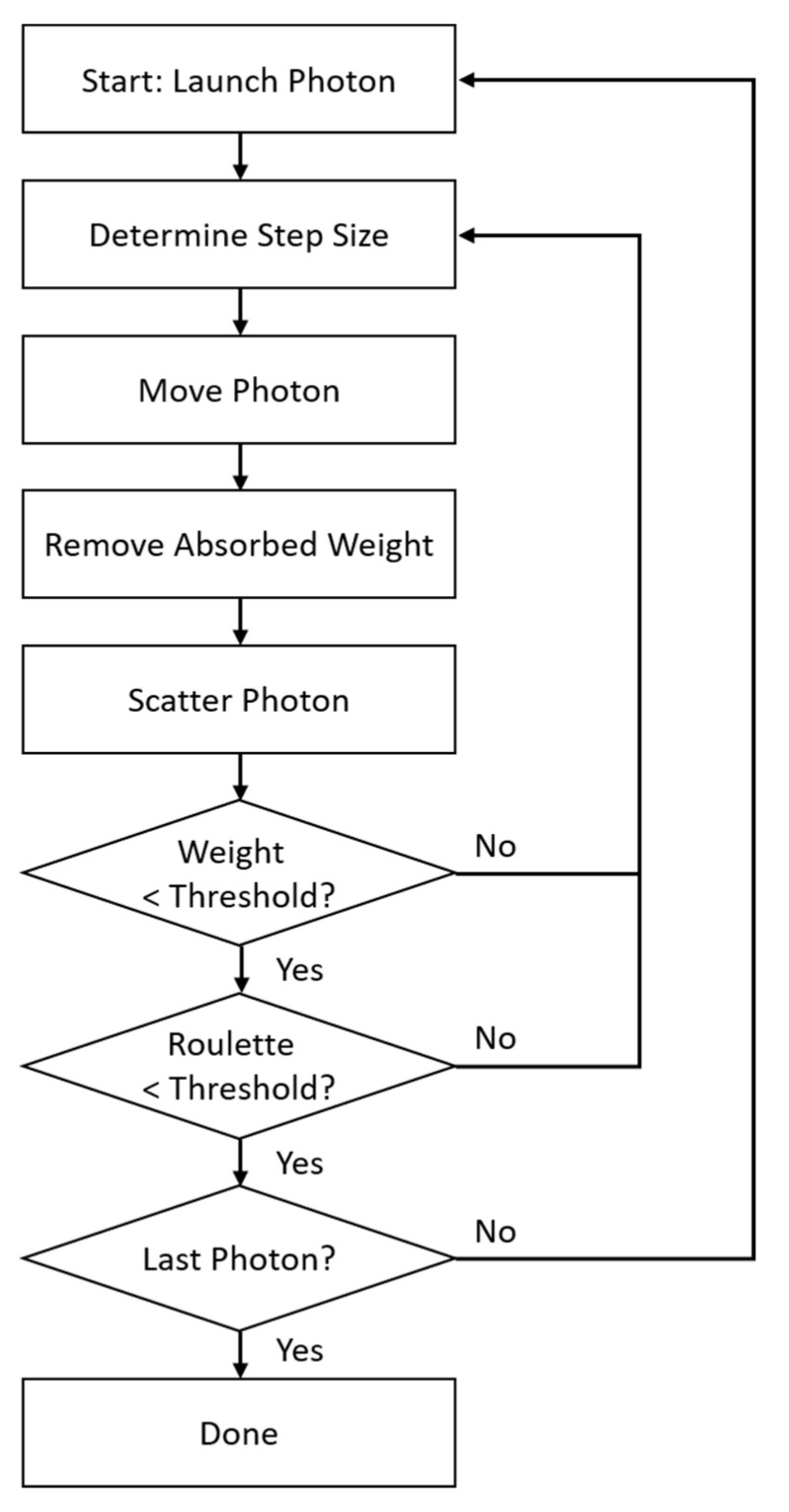
Monte Carlo simulation flow chart.

The power of MCS lies in its flexibility. After setting up the source(s), detector(s), and model, the simulation process emits one photon at a time and interacts with the model. Figure 9 shows how this simulation works. First, a photon is generated from the position of the source with a random direction with a predefined distribution. Initially, the photon weight is set as 1. The step size is related to the total extinction (absorption) coefficient of the medium. Then, the photon is moved to the direction with the calculated step. The absorbed weight is then calculated, and its value is removed from the photon. This is the application of the absorption coefficient of the medium. The new direction of the photon is calculated using the scattering coefficient of the medium. Next, the photon is checked for the weight threshold, the roulette threshold. The roulette is performed to conserve the total energy of the system. If a photon exceeds the weight and roulette thresholds, the photon is again traversed from the “step size” stage. In this simulation, if any photon passes the region near the detector position, the photon ID and weight are recorded. If the last photon is simulated, the simulation stops. Figure 9 shows the Monte Carlo simulation flow chart. An examples of the MCS table can be found in Supplementary Table S1.

**Figure 10:**
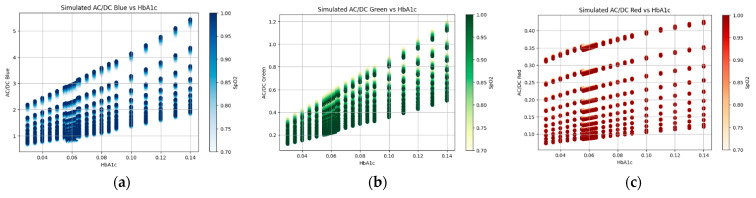
(**a**) Simulated AC/DC (blue) vs. HbA1c, (**b**) simulated AC/DC (green) vs. HbA1c, and (**c**) simulated AC/DC (red) vs. HbA1c with variable SpO2 values.

In Figure 10, simulated AC/DC values vs. HbA1c are visualized with variable SpO2 values. From the figure, it is clear that AC/DC values increase with HbA1c. Here, the AC/DC values were calculated using Equation (11). In (11), $I_{PPG}^{dia}$ represents the PPG intensity during diastole (heart relaxation) and $I_{PPG}^{sys}$ represents the PPG intensity during systole (heart contraction). The numerator, $I_{PPG}^{dia}-I_{PPG}^{sys}$, captures the pulsatile change in blood volume, while the denominator, $I_{PPG}^{sys}$, represents the baseline blood volume. The resulting ratio quantifies the relative change in blood volume due to heartbeats.

(11) $$\frac{AC}{DC}=\frac{I_{PPG}^{sys}-I_{PPG}^{dia}}{I_{PPG}^{dia}}$$

**Figure 11:**
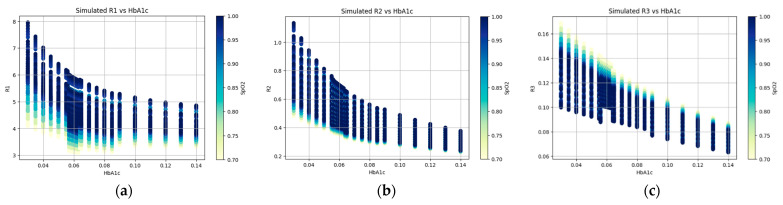
(**a**) Simulated R1 vs. HbA1c, (**b**) simulated R2 vs. HbA1c, and (**c**) simulated R3 vs. HbA1c with variable SpO2 values.

In Figure 11, the simulated ratio vs. HbA1c values are visualized with variable SpO2 values. The ratio values (R1, R2, and R3) were calculated using Equations (8)–(10). From the figure, it is clear that the ratio values decrease exponentially with HbA1c. The color bar also represents the range of SpO2 values. The visualization of cylindrical intensity versus melanin (or bilirubin) values at blue, green, and red wavelengths can be found in Supplementary Figure S3 (or Figure S4).

The Monte Carlo simulation results in Figure 10 and Figure 11 confirm that there is a nearly linear relationship between the PPG signal and HbA1c. In addition, it can be seen in Figure 6 that the relationship between the actual PPG signal and HbA1c shows similar results to those in the Monte Carlo simulation. This confirms a clear relationship between the PPG signal and HbA1c.

### 2.5. Calibration Using XGBoost Regression

**Figure 12:**
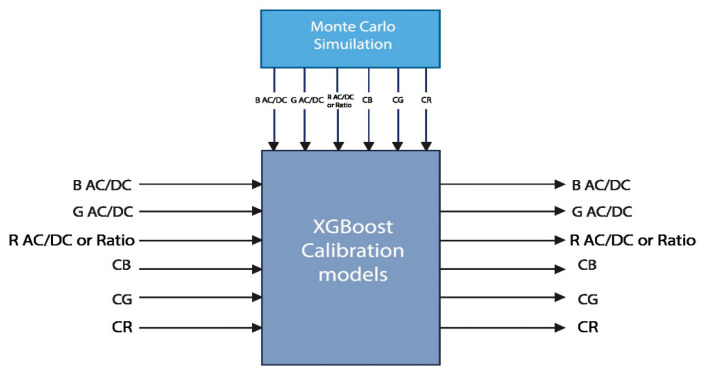
XGBoost calibration model.

The intensity values from the Monte Carlo simulation have different scales to the PPG signals acquired from a wrist-type device because the source is set to the weight of 1 in the simulation. For this reason, the PPG signals received from the device and the received intensity values from MCS need to be calibrated. Note that the skin effect was not considered in our previous study [9]. However, the skin effect plays a large role in noninvasive in vivo estimation tasks. Therefore, in this study, we considered additional cylindrical signals during calibration and testing to reduce skin color-related errors in the estimation process. For calibration, this study used the XGBoost regression model shown in Figure 12. Here, we can see that the XGBoost calibration model has 6 inputs and 6 outputs.

First (Method 1), calibration was performed by considering three (blue, green, and red) AC/DC values and three (blue, green, and red) cylindrical intensity values. The target values were obtained through Monte Carlo simulation. Secondly (Method 2), a similar approach was followed using two (blue and green) AC/DC values, one ratio (blue/green), and three (blue, green, and red) cylindrical intensity values.

## 3. Results and Discussion

After Monte Carlo simulations for a total of 69,212 times depending on the range of HbA1c (3% to 14%), SpO2 (70% to 100%), systolic/diastolic (0/1), melanin (1% to 20%), and bilirubin (0.1% to 20%), we received different intensities of light at the designated receivers for the watch-type model. For HbA1c, 0.5% intervals were applied in the ranges of 3.0% to 5.0% and 6.5% to 9%, 0.1% intervals were applied in the range of 5.0% to 6.5%, and 1% intervals were applied in the range of 9% to 14%. For SpO2, 5% intervals were applied in the range of 70% to 95%, and 1% intervals were applied in the range of 95% to 100%. For systolic/diastolic, systolic was applied as 0 and diastolic was applied as 1. For melanin and bilirubin, an interval of 2% was applied. This resulted in a total of 69,212 combinations of HbA1c (26), SpO2 (11), systolic/diastolic (2), melanin (11), and bilirubin (11). The reason for applying different intervals for each specific section is to optimize the simulation time for MCS while simulating critical sections (e.g., 5.0% to 6.5% for HbA1c) in more detail. In addition, it was concluded that changes in bilirubin did not affect the results, so the experiment was conducted by fixing it at a value of 0.121.

### 3.1. Method 1: Using 3 AC/DC and 3 Cylindrical Values

**Figure 13:**
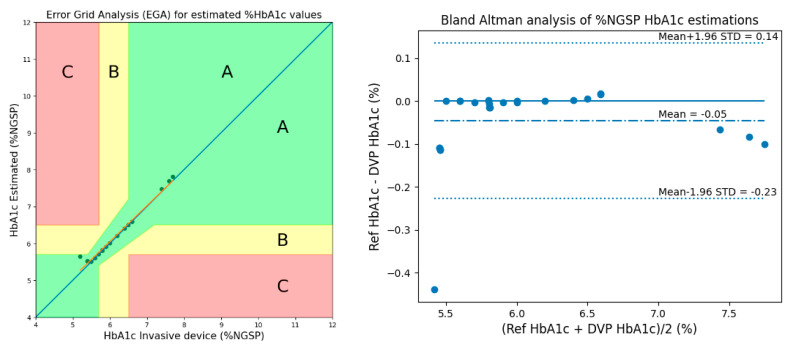
EGA and Bland–Altman analysis for HbA1c considering 3 AC/DCs and 3 cylindrical values.

After calibrations were performed, Clarke’s error-grid analysis (EGA) [14] and Bland–Altman analysis plots were used to evaluate the performance of the estimated HbA1c values. As shown in Figure 13 of the EGA, Zone A (clinically accurate) contains 28 samples (100 percent), Zone B (data outside of 20 percent of the reference but that would not lead to inappropriate treatment) contains 0 samples (0 percent), and Zone C (data that would lead to inappropriate treatment) contains 0 samples (zero percent). According to the Bland–Altman analysis, the bias was −0.0456 ± 0.0924, while the limits of agreement ranged from −0.2267 to 0.1354.

Table 2 presents the performance statistics for estimating HbA1c levels using Method 1. The estimation method shows high accuracy and precision, as evidenced by a low MSE (0.0106), low ME (−0.0456), and low MAD (0.0490). Additionally, there is a strong positive linear relationship between the estimated and actual values (Pearson’s r = 0.9907). The accuracy is 99.14%, indicating a high level of accuracy in the estimation process.

### 3.2. Method 2: Using 2 AC/DCs, 1 Ratio, and 3 Cylindrical Values

**Figure 14:**
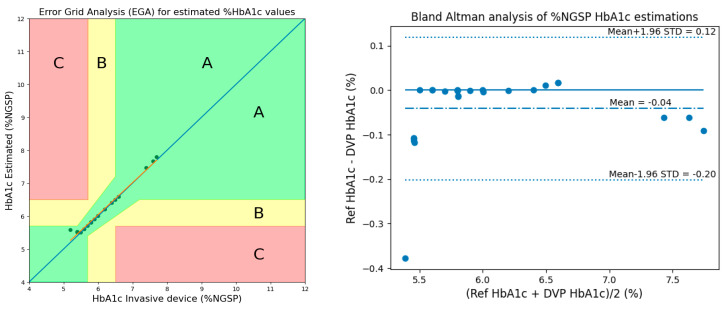
EGA and Bland–Altman analysis for HbA1c estimation considering 2 AC/DCs, 1 ratio, and 3 cylindrical values.

Similarly, performance was evaluated using Clarke’s EGA [14] and Bland–Altman analysis plots while considering 2 AC/DCs, 1 ratio, and 3 cylindrical values for calibration. As shown in Figure 14 of the EGA, Zone A contains 28 samples similar to Method 1, Zone B contains 0 samples, and Zone C contains 0 samples. According to the Bland–Altman analysis, the bias of the blood-vessel model was −0.0416 ± 0.0818, while the limits of agreement ranged from −0.2018 to 0.1187.

Table 3 presents performance statistics for estimating HbA1c levels using 2 AC/DCs, 1 ratio, and 3 cylindrical values. The estimation results show improved performance compared to Method 1 in the previous section, with lower MSE (0.0084), lower MAD (0.0451), lower RMSE (0.0917), and higher Pearson’s r (0.9928). RMSE (0.0917) indicates a narrow estimation error range and is slightly lower than Method 1. The accuracy of 99.21% is also slightly higher than Method 1, showing better performance.

From the results, we can see that the results of Method 2 are better than those of Method 1. This is because Method 1 considered red AC/DC values. However, the red PPG signal was not as good, resulting in worse AC/DC values. The result occurred because the absorption rate of hemoglobin decreases as the wavelengths [15]. This is why this study excluded the red PPG signal in Method 2 and instead used the ratio of blue and green AC/DC values.

### 3.3. Comparison with Previous Studies

In our previous study [9], HbA1c was estimated without considering skin effects. Using our proposed methods (Method 1 and Method 2), the HbA1c estimation performance of HbA1c was better than the previous method in terms of metrics (MSE, MAD, RMSE, and Pearson’s r). Only one metric, ME, was slightly worse than the previous method. Apart from that, all other metrics showed strong performance improvements compared to the previous study. In the case of ME, it is the sum of all error values divided by the number of records. Therefore, if positive and negative errors are mixed, they cancel each other out in the calculation, resulting in a low value. For this reason, the ME performance of Shifat et al. [9] was better than the proposed method. Checking the EGA plot, we found that Shifat et al. [9] spread both positive and negative errors compared to the proposed method. The comparison of HbA1c estimation performance between this study and the previous study [9] can be seen in Table 4.

### 3.4. Result Using 50 Subjects’ Data

**Figure 15:**
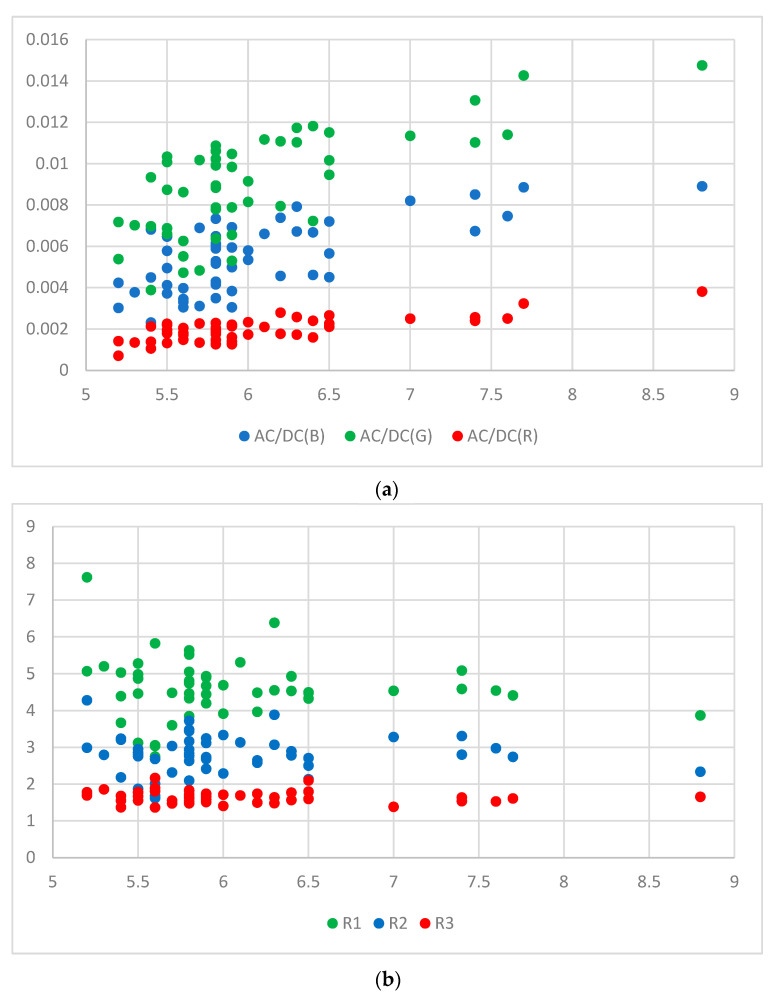
(**a**) AC/DC versus HbA1c for 50 subjects, (**b**) Ratio versus HbA1c for 50 subjects.

In order to expand the distribution of data and increase the reliability of estimation accuracy, an experiment was conducted by increasing the number of subjects to 50. The analysis was performed using data collected from 50 subjects under the supervision of the institutional review board (IRB) of Kookmin University (IRB protocol number: KMU-202111-BR-286). The participants’ ages ranged from 25 to 80 years. Of them, 15 subjects (30%) were pre-diabetic, 9 subjects (18%) were diabetic, and 26 subjects (52%) were healthy. Figure 15 shows AC/DC values versus HbA1c and ratio versus HbA1c for the 50 subjects.

**Figure 16:**
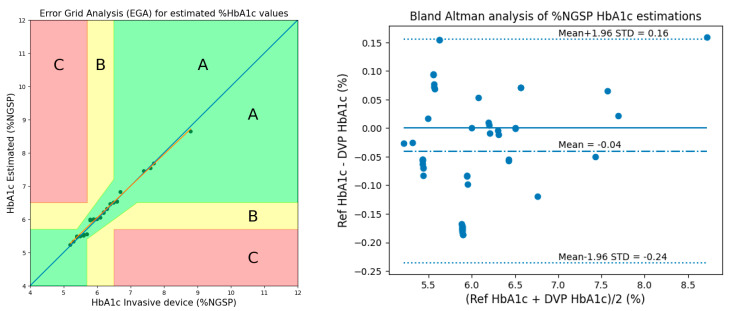
EGA and Bland–Altman analysis for HbA1c considering 3 AC/DCs and 3 cylindrical values in 50 subjects.

The performance of the proposed methods was evaluated using Clarke’s EGA and Bland–Altman analysis. Figure 16 shows the results of EGA and Bland–Altman analysis for HbA1c considering 3 AC/DCs and 3 cylindrical values in 50 subjects.

**Figure 17:**
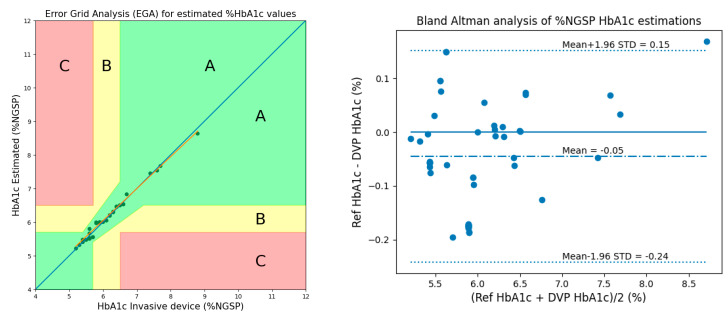
EGA and Bland–Altman analysis for HbA1c when considering 2 AC/DCs, 1 ratio, and 3 cylindrical values (Method 2) in 50 subjects.

Figure 17 shows the results of EGA and Bland–Altman analysis for HbA1c when considering 2 AC/DCs, 1 ratio, and 3 cylindrical values (Method 2) in 50 subjects.

Table 5 presents a comparison with our previous study on HbA1c estimation performance. The results for 50 subjects showed slightly lower performance than the results for 28 subjects. However, our method still showed better performance compared to that of Shifat et al. [9] and showed a similar trend to the results of 28 subjects. The reason for the slightly lower performance of the results with 50 subjects can be seen as a natural decrease due to the increase in data distribution.

Therefore, it can be said that the proposed method considering the skin effect shows better performance than the existing method in [9]. In addition, it was confirmed that sufficiently reliable results could be obtained even if the number of subjects was increased to 50.

### 3.5. Discussion

Due to skin effects that were not considered in the previous study [9], inaccurate results can occur. To address this, we added skin effects to the Monte Carlo simulation to achieve more accurate estimation. Additionally, an optical barrier was installed between the LED and the PD to effectively remove intrinsic and extrinsic noise, and we added a cylindrical sensor to remove the skin effect noise from the PPG signal by capturing the skin reflection signal at a certain distance. The proposed method shows superior performance over the previous method and shows better performance indicators such as lower MSE, and MAD and a higher Pearson’s r value. These improvements were also reflected in Clarke’s EGA and Bland–Altman analysis, indicating strong agreement between estimated and reference HbA1c values. Furthermore, 22 subjects were added to further validate the performance of the proposed method for a total of 50 subjects. Although the performance was slightly decreased compared to the existing 28 subjects, this is believed to be due to the increase in data distribution. This confirmed that the results were sufficiently reliable even when the number of subjects was increased, showing better performance than the previous study in [9].

The proposed method, especially method 2, demonstrated better accuracy and consistency in HbA1c estimation compared to the previous method. As shown in Table 5, it was confirmed that the performance of the proposed method was improved. In particular, in terms of Pearson’s r values, the proposed Method 1 (28 subjects) showed an increase of 0.0207 compared to the result (0.97) of Shifat et al.’s [9] method, and the proposed Method 2 (28 subjects) showed an increase of 0.0228, confirming the significant improvement in performance. The results of performance statistics based on various error metrics including Pearson’s r indicate that the proposed method outperforms the existing methods. In addition, to ensure the reliability of the results, the number of subjects was increased from 28 to 50. Based on Pearson’s r, the value for 50 subjects was 0.9894, an increase of 0.0194 from the Pearson’s r value in [9], and a decrease of 0.0013 and 0.0033 compared to the results of 28 subjects for the proposed methods, Method 1 and Method 2, but this is fully acceptable because the data distribution increased. This shows that sufficient performance can be obtained even if the number of subjects increases, suggesting that accurate and reliable measurements for noninvasive glycated hemoglobin measurements can be provided.

To address the challenges associated with reliability in noninvasive measurements, we increased the number of test subjects from 28 to 50 as well as considering the skin effect. In addition, the reliability of calibration was improved by finding parameters (AC to DC ratio, ratio of ratios, etc.) that determine the relationship with HbA1c from the PPG signal. In related research fields, about 30 subjects are usually used, but we targeted 50 subjects in order to conduct research on as many subjects as possible. This provided enough evidence of having clear trends between physiological signal (PPG) and HbA1c estimation results. From our Monte Carlo simulation (MCS) as well, we obtained clear trends in AC/DC, ratio of ratios, and HbA1c levels. Therefore, based on the PPG signal results and MCS results, we can say that there is a good trend between physiological signals and HbA1c levels. The analysis was performed using a dataset consisting of a total of 50 subjects under the direct supervision of the IRB of Kookmin University (IRB protocol number: KMU-202111-BR-286), Seoul, Republic of Korea. The number and diversity of subjects can inevitably be limited due to the nature of targeting human biosignals. To overcome this, we plan to increase the number of subjects through clinical trials in cooperation with large hospitals in the near future to guarantee the size and diversity of the sample.

We have also been working on minimizing the influence of the data collection environment. The experimental environment (temperature, humidity, brightness, etc.) was kept the same during the measurement. And we tried to control motion artifacts, which are the most representative examples of variables that affect the results depending on these experimental environments. Due to the nature of collecting data through wrist-type devices, PPG signals can be erroneous due to wrist or hand movements. To prevent this, motion artifacts are currently minimized by strictly controlling the movement of subjects. Additionally, we are currently studying how to isolate and remove motion artifacts from the PPG signal, thereby obtaining error-free signals without controlling the experimental environment. This is expected to improve the accuracy and reliability of HbA1c estimates, and the results will be published in the near future.

## 4. Conclusions

In this study, we successfully developed and validated a Monte Carlo simulation model for noninvasive HbA1c estimation considering skin effects. In order to consider skin effects in Monte Carlo simulation, a new simulation was performed by additionally considering melanin, bilirubin, and cylindrical sensor signals to the existing wrist model. Through this, a total of 69,212 combinations were simulated to complete MCS considering skin effects. To apply the same situation as the simulation, a hardware configuration was designed and implemented to remove intrinsic and extrinsic noise using an optical barrier, and a cylindrical sensor was added to complete the device, taking skin effects into account.

In this study, we improved the noninvasive glycated hemoglobin measurement performance considering skin effects and confirmed that the proposed noninvasive method can be an appropriate replacement for the invasive state-of-the-art method. However, the proposed method may still have issues to be addressed. A typical example is motion artifact removal. Due to the nature of collecting data through a wrist-type device, errors may occur in the PPG signal due to wrist or hand movements. As a future study, we aim to secure stable performance by considering additional parameters (motion artifacts, etc.) that can be sources of errors.

## Figures and Tables

**Table 1:** Statistics of the measured %HbA1c data.

	Min	Max	Mean	Median	SD	25th Percentile	75th Percentile
%HbA1c	5.20	7.70	6.04	5.80	0.678	5.60	6.40

**Table 2:** HbA1c estimation performance statistics considering 3 AC/DCs and 3 cylindrical values.

	MSE	ME	MAD	RMSE	Pearson’s r	%Accuracy
Proposed (Method 1, 28 subjects)	0.0106	−0.0456	0.0490	0.1030	0.9907	99.14

MSE: mean square error, ME: mean error, MAD: mean absolute deviation, and RMSE: root mean square error.

**Table 3:** HbA1c estimation performance statistics considering 2 AC/DCs, 1 ratio, and 3 cylindrical values.

	MSE	ME	MAD	RMSE	Pearson’s r	%Accuracy
Proposed (Method 2, 28 subjects)	0.0084	−0.0416	0.0451	0.0917	0.9928	99.21

**Table 4:** HbA1c estimation performance statistics considering 2 AC/DCs, 1 ratio, and 3 cylindrical values.

	Metric	MSE	ME	MAD	RMSE	Pearson’s r
Method						
Shifat et al. [9] (28 subjects)		0.0500	0.0100	0.1300	0.2100	0.9700
Proposed (Method 1, 28 subjects)		0.0106	0.0456	0.0490	0.1030	0.9907
Proposed (Method 2, 28 subjects)		0.0084	0.0416	0.0451	0.0917	0.9928

**Table 5:** HbA1c estimation performance statistics considering 2 AC/DCs, 1 ratio, and 3 cylindrical values.

	Metric	MSE	ME	MAD	RMSE	Pearson’s r
Method						
Shifat et al. [9] (28 subjects)		0.0500	0.0100	0.1300	0.2100	0.9700
Proposed (Method 1, 28 subjects)		0.0106	0.0456	0.0490	0.1030	0.9907
Proposed (Method 2, 28 subjects)		0.0084	0.0416	0.0451	0.0917	0.9928
Proposed (Method 1, 50 subjects)		0.0116	0.0402	0.0877	0.1075	0.9894
Proposed (Method 2, 50 subjects)		0.0121	0.0451	0.0887	0.110	0.9894

## Data Availability

The dataset used in this research is available upon valid request to any of the authors of this research article.

## Supplementary Materials

The following supporting information can be downloaded at: https://www.mdpi.com/article/10.3390/mi15091067/s1. Figure S1: Histograms of the measured dataset: %NGSP HbA1c values for 28 subjects; Figure S2: Histograms of the measured dataset: %NGSP HbA1c values for 50 subjects; Figure S3: Visualization of Cylindrical intensity values vs. Melanin (CB: Cylindrical Blue, CG: Cylindrical Green, CR: Cylindrical Red): (a) CB vs. Melanin, (b) CG vs. Melanin, and (c) CR vs. Melanin; Figure S4: (a) HbA1c vs. Bilirubin, (b) Melanin vs. Bilirubin, (c) Simulated Blue AC/DC vs. Bilirubin, and (d) Simulated ratio vs. Bilirubin; Table S1: MCS table example considering skin effects.
