# Supplementary material for: Design and Validation of a Monte Carlo Method for the Implementation of Noninvasive Wearable Devices for HbA1c Estimation Considering the Skin Effect
Source: Micromachines (Basel). 2024 Aug 24;15(9):1067. doi: 10.3390/mi15091067 (PMC11434557; doi:10.3390/mi15091067)
Supplement: Supplementary file 1 [file micromachines-15-01067-s001.zip › micromachines-3108989-supplementary.pdf]

Supporting information

Design and Validation of Monte Carlo Method to Implement in Noninvasive Wearable Device for HbA1c Estimation Considering Skin Effect

Tae-Ho Kwon[1], Shifat Hossain[2], Mrinmoy Sarker Turja[1] and Ki-Doo Kim[1,*]

[1] Department of Electronics Engineering, Kookmin University, Seoul 02707, Republic of Korea; kmjkth@kookmin.ac.kr
[2] Department of Electrical and Computer Engineering, University of Central Florida, Orlando, FL 32816, USA; shifathosn@gmail.com
* Correspondence: kdk@kookmin.ac.kr; Tel.: +82-2-910-4707

Supplementary Sections

Figure S1: Histograms of the measured dataset: %NGSP HbA1c values for 28 subjects.

Figure S2: Histograms of the measured dataset: %NGSP HbA1c values for 50 subjects.

Figure S3: Visualization of Cylindrical intensity values vs Melanin (CB: Cylindrical Blue, CG: Cylindrical Green, CR: Cylindrical Red) (a) CB vs Melanin, (b) CG vs Melanin, (c) CR vs Melanin.

Figure S4: (a) HbA1c vs Bilirubin, (b) Melanin vs Bilirubin, (c) Simulated Blue AC/DC vs Bilirubin, (d) Simulated ratio vs Bilirubin.;

Table S1: MCS table example considering skin effect.

Supplementary Materials

Figure S1 shows the distribution of %NGSP HbA1c values for 28 subjects.

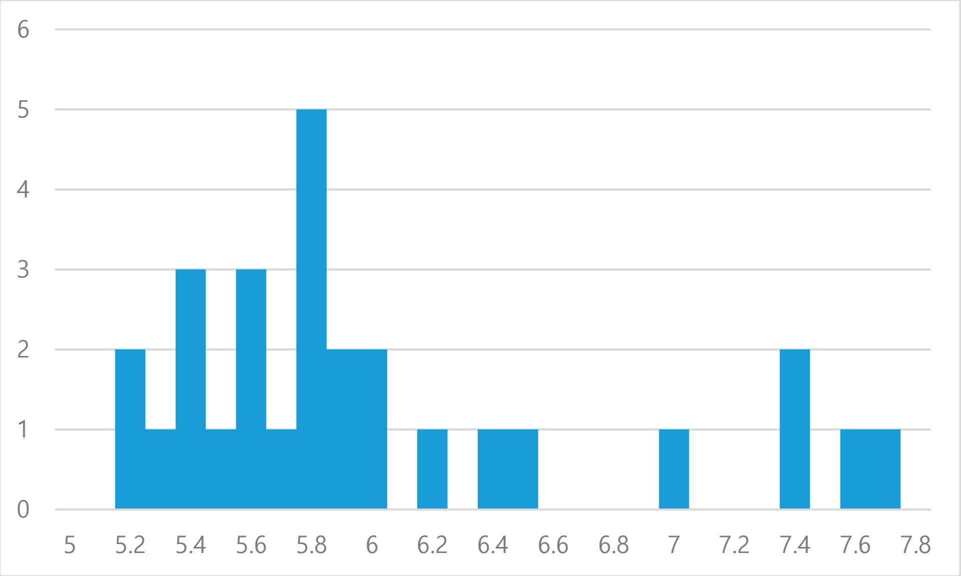

Figure S1. Histograms of the measured dataset: %NGSP HbA1c values for 28 subjects.

Figure S2 shows the distribution of %NGSP HbA1c values for the 50 subjects.

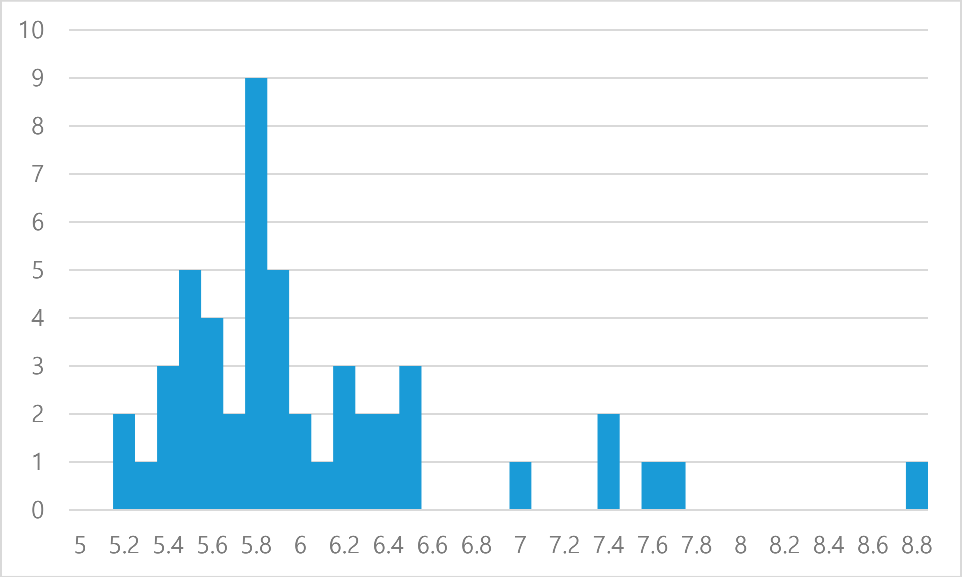

Figure S2. Histograms of the measured dataset: %NGSP HbA1c values for 50 subjects.

Figure S3 presents a visualization of cylindrical intensity versus melanin values at blue, green, and red wavelengths. We can see that the cylindrical value decreases as the melanin level increases. Actually, the cylindrical signal is a reflective signal. Therefore, darker skin colors (higher melanin) will result in lower reflected signals, while lighter skin colors (lower melanin) will result in higher reflected signals.

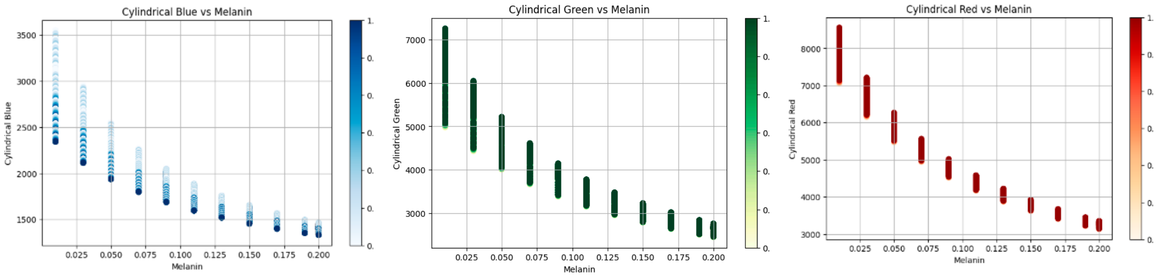

(a) **(b)** **(c)**

Figure S3. Visualization of Cylindrical intensity values vs Melanin (CB: Cylindrical Blue, CG: Cylindrical Green, CR: Cylindrical Red) (a) CB vs Melanin, (b) CG vs Melanin, (c) CR vs Melanin.

Figure S4 shows an overall visualization of the effect of bilirubin on HbA1c, melanin, AC/DC, and ratio (R) values. This makes it clear that bilirubin literally has no effect on any of these. Therefore, this study did not consider all bilirubin values and only used a constant bilirubin level of 0.121 for convenience. This value was chosen randomly. Through this, the computation time was also greatly reduced.

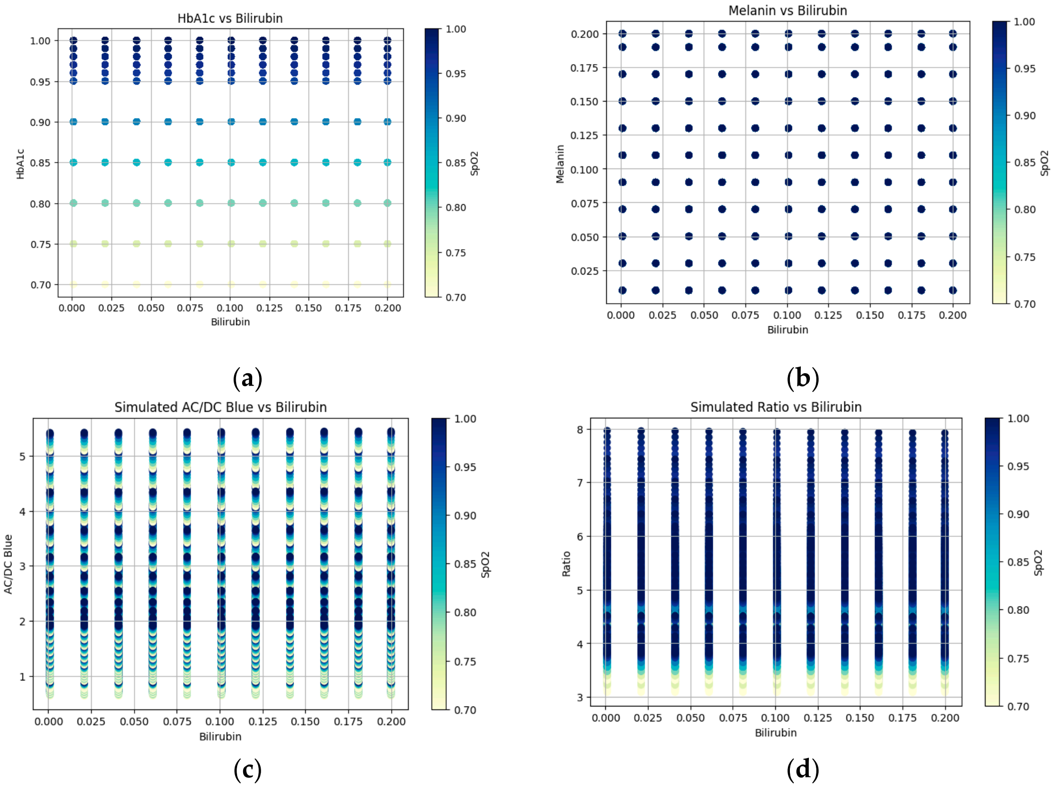

Figure S4. (a) HbA1c vs Bilirubin, (b) Melanin vs Bilirubin, (c) Simulated Blue AC/DC vs Bilirubin, (d) Simulated ratio vs Bilirubin.

The table generated from the simulation has headings: HbA1c, SpO2, Melanin(Mel), Bilirubin(Bil), Systolic(Sys(0)) and Diastolic(Dias(1)), PPG signal intensity (B, G, R), cylindrical signal intensity (CB, CG, CR), respectively. Table S1 shows an examples of the MCS table.

Table S1. MCS table example considering skin effect.

HbA1c	SpO2	Mel	Bil	Sys(0)/ Dias(1)	B	G	R	CB	CG	CR
0.03	0.7	0.01	0.001	1	103.184	1431.179	3885.104	3535.071	6865.962	8260.994
0.03	0.7	0.01	0.001	0	299.822	1937.794	5106.585	4554.178	7573.247	8632.782
0.03	0.7	0.03	0.001	1	42.531	813.104	3375.171	2939.976	5788.596	7007.803
0.03	0.7	0.03	0.001	0	105.193	1058.455	4208.187	3550.182	6256.674	7260.889
					.					
					.					
					.					
0.14	1	0.19	0.2	1	0.098	21.708	1647.755	1351.8	2536.573	3245.379
0.14	1	0.19	0.2	0	0.291	32.866	1857.568	1476.744	2733.718	3371.881
0.14	1	0.2	0.2	1	0.078	18.901	1619.849	1330.437	2476.438	3158.847
0.14	1	0.2	0.2	0	0.230	28.391	1816.381	1446.457	2661.069	3277.275
